# A purely endoscopic management approach for Type V Mirizzi syndrome

**DOI:** 10.1016/j.vgie.2021.05.007

**Published:** 2021-06-09

**Authors:** Sarah S. Al Ghamdi, Michael Bejjani, Bachir Ghandour, Mouen A. Khashab

**Affiliations:** Division of Gastroenterology and Hepatology, Johns Hopkins Hospital, Baltimore, Maryland

**Keywords:** CCF, cholecystocolonic fistula, CD, cystic duct, EHL, electrohydraulic lithotripsy, GB, gallbladder, MS, Mirizzi syndrome

## Abstract

Video 1 A purely endoscopic management approach for Type V Mirizzi syndrome.

A purely endoscopic management approach for Type V Mirizzi syndrome.

## Background

A cholecystocolonic fistula (CCF) is a communication between the gallbladder (GB) and the colon. When it coexists with any type of Mirizzi syndrome (MS), this is referred to as Type V MS.^1^ When it is symptomatic, surgical treatment with cholecystectomy, fistula takedown, and possible colonic resection is indicated.^2^^,^^3^ The role of treatment in asymptomatic patients is unclear. Endoscopic management has not been described.

## Case presentation

A 94-year-old female with a history of type 2 diabetes mellitus, hypertension, and hypothyroidism was admitted to an outside hospital with a mechanical fall and acute kidney injury. On day 5 of admission, she developed acute cholangitis. MRCP revealed a large calcified gallstone in the cystic duct (CD) with common hepatic duct and intrahepatic duct dilation. The next day, she underwent urgent ERCP for hemodynamic instability. Cholangiogram revealed intrahepatic duct dilation with many large stones in the CD and GB. A plastic biliary stent was placed. Sphincterotomy was not performed because of a prolonged international normalized ratio of 1:9. Several days later, she was transferred to our hospital for further management.

## Endoscopic methods

Repeat ERCP was performed (Video 1, available online at www.giejournal.org). Cholangiogram revealed a dilated CD with multiple large CD stones. The common bile duct was compressed externally from the stones, and there was evidence of common hepatic duct and intrahepatic duct dilation, consistent with Type I MS. Because of the large stone burden, cholangioscopy was performed, which revealed a large impacted stone at the CD takeoff.

**Figure 1:**
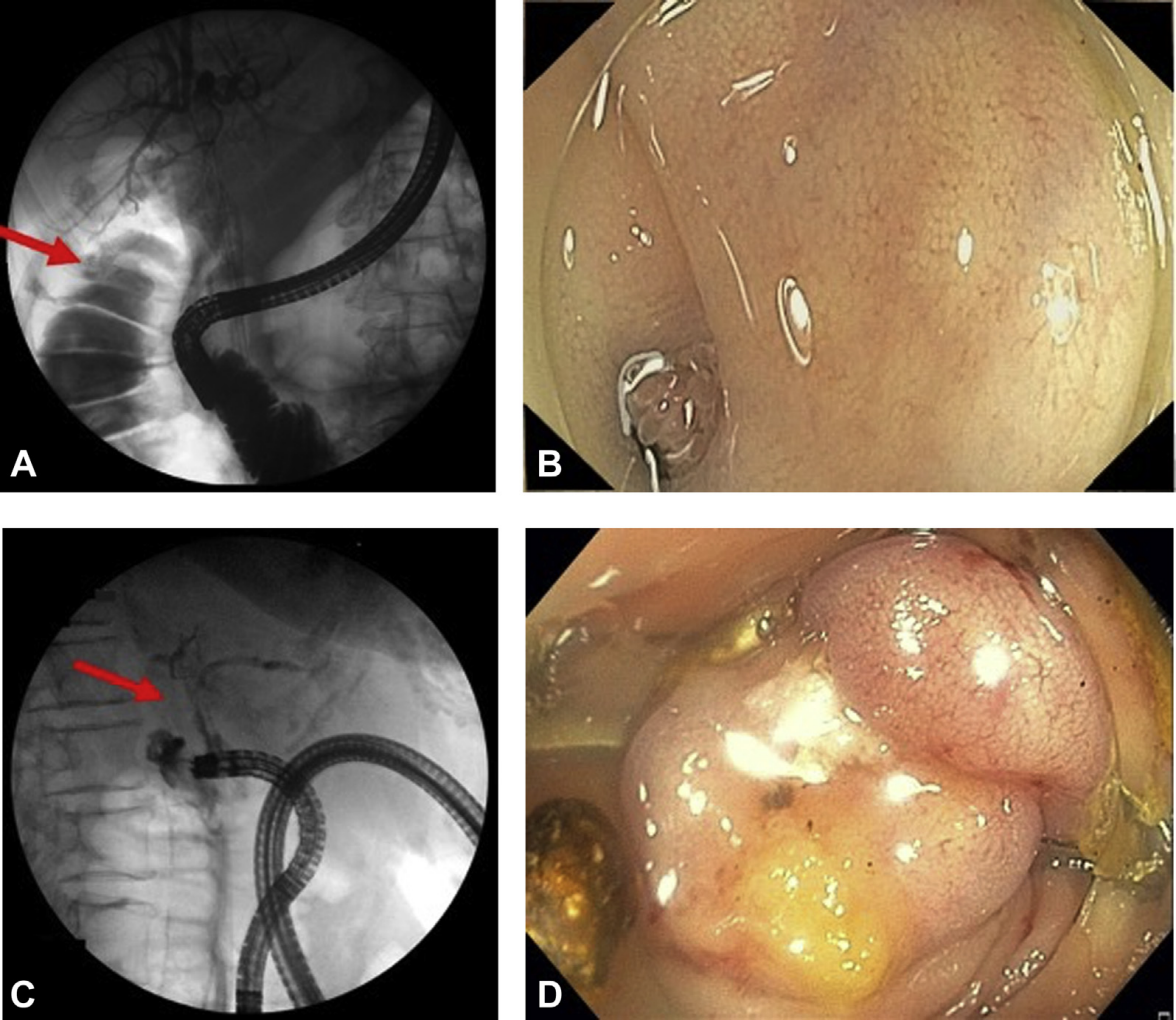
**A,** Occlusion cholangiogram revealed extravasation of contrast from the gallbladder into the colon, suggestive of a cholecystocolonic fistula. **B,** Small mucosal defect with intermittent extrusion of microvilli was seen at the hepatic flexure. **C,** Contrast was injected into the defect, confirming correct location of the cholecystocolonic fistula. **D,** A 14-mm/6-mm type t over-the-scope clip was deployed over the fistula.

Electrohydraulic lithotripsy (EHL) was performed to fragment the stone. Occlusion cholangiogram revealed residual stone in the CD, which had significantly decreased in size, and revealed extravasation of contrast from the GB into the colon, suggestive of a CCF (Fig. 1A). This was diagnostic of Type V MS.

Colonoscopy was performed 1 week later to evaluate the fistula. Several small gallstones were seen in the colon, and a small mucosal defect with intermittent extrusion of microvilli was seen at the hepatic flexure (Fig. 1B). Contrast was injected into the defect, confirming correct location of the CCF (Fig. 1C). The fistula tract was deepithelialized using argon plasma coagulation, and an endoclip was placed to mark the area.

A 14-mm/6-mm type t over-the-scope clip was deployed over the fistula (Fig. 1D). Contrast injection confirmed complete closure of the fistula. After the procedure, the patient's abdominal pain resolved completely.

The patient returned 5 months later to undergo her final ERCP. Cholangioscopy revealed a residual stone at the CD takeoff, which was treated with EHL. Occlusion cholangiogram showed no further filling defects in the biliary tree, confirming complete stone clearance. At 6-month follow-up, the patient was doing well with normal liver chemistries and no symptoms.

## Conclusions

This case demonstrates the use of ERCP and EHL for successful management of a large impacted CD stone resulting in biliary obstruction and acute cholangitis. Endoscopic management of a concomitant CCF using an over-the-scope clip was also successful. This suggests the safety and efficacy of endoscopic management as a minimally invasive option for Type V MS.

## Disclosure

*Dr Khashab is a consultant for Boston Scientific, Olympus, Triton, Medtronic, and GI Supply. All other authors disclosed no financial relationships.*

## Supplementary data

Video 1 A purely endoscopic management approach for Type V Mirizzi syndrome.

## References

[1] Bonventre G., Di Buono G., Buscemi S. (2020). Laparoscopic management of cholecystocolonic fistula: a case report and a brief literature review. Int J Surg Case Rep.

[2] Yetişir F., Şarer A.E., Acar H.Z. (2016). Laparoscopic resection of cholecystocolic fistula and subtotal cholecystectomy by tri-staple in a Type V Mirizzi Syndrome. Case Reports Hepatol.

[3] Li X.Y., Zhao X., Zheng P. (2017). Laparoscopic management of cholecystoenteric fistula: a single-center experience. J Int Med Res.

